# Transspinal Direct Current Electrical Stimulation Selectively Affects the Excitability of the Corticospinal System, Depending on the Intensity but Not Motor Skills

**DOI:** 10.3390/life13122353

**Published:** 2023-12-16

**Authors:** Alena Popyvanova, Ekaterina Pomelova, Dmitry Bredikhin, Maria Koriakina, Anna Shestakova, Evgeny Blagovechtchenski

**Affiliations:** Centre for Cognition and Decision Making, Institute for Cognitive Neuroscience, HSE University, 101000 Moscow, Russia; apopyvanova@hse.ru (A.P.); epomelova@hse.ru (E.P.); dbredihin@hse.ru (D.B.); mkoriakina@hse.ru (M.K.); a.shestakova@hse.ru (A.S.)

**Keywords:** non-invasive brain stimulation, transspinal stimulation, transcranial magnetic stimulation, motor skills

## Abstract

Transspinal direct current stimulation (tsDCS) is a non-invasive technique used to modulate spinal cord activity. However, the effects and mechanisms of this stimulation are currently not comprehensively known. This study aimed to estimate the effect of different intensities of tsDCS applied at the level of cervical enlargement of the spinal cord (C7-Th1 segments) on the excitability of the corticospinal system (CSS) and the correction of motor skills in healthy subjects. The effect of tsDCS was estimated by the motor-evoked potentials (MEP) elicited by transcranial magnetic stimulation (TMS) in the primary motor cortex (M1). The study involved 54 healthy adults aged 22 ± 4 years. The application of 11 min anodal tsDCS at the level of the cervical spine C7-Th1 with a current intensity of 2.5 mA did not change the MEP amplitude of the upper limb muscles, in contrast to the data that we previously obtained with a current intensity of 1.5 mA. We also found no difference in the effect of 2.5 mA stimulation on motor skill correction in healthy subjects in the nine-hole peg test (9-HPT) and the serial reaction time task (SRT) as with 1.5 mA stimulation. Our data show that an increase in the intensity of stimulation does not lead to an increase in the effects but rather reduces the effects of stimulation. These results provide information about the optimally appropriate stimulation current intensities to induce CSS excitability and the ability of tsDCS to influence motor skills in healthy adults.

## 1. Introduction

The corticospinal system (CSS) is one of the central systems involved in the control of precise voluntary movements; therefore, one can expect effects associated with the activity of the muscles involved in implementing the patterns of such movements [1]. This study aimed to estimate the effect of the intensity parameters of anodal transspinal direct current stimulation (tsDCS), which is a non-invasive technique with a neuromodulatory effect on the spinal circuits associated with the motor and sensory responses of the upper and lower extremities [2,3], applied at the level of cervical enlargement of the spinal cord, on the activity of the corticospinal system and the correction of the fine motor skills of the upper limbs. 

In the current study, we performed a statistical comparison of our previous data with 1.5 mA tsDCS, where the results showed that the application of anodal tsDCS at 1.5 mA affects the muscles of the upper limbs, first reducing the amplitude of TMS-induced MEP immediately after stimulation, and, 15 min after stimulation, the amplitude of MEP increases. The literature shows a large range of effects of tsDCS, from significant to completely absent (see below). One explanation is that the differences in effects may be due to the differences in the study protocols used, which have variable factors, such as the stimulation polarity, electrode placement, and current strength [4]. It is interesting to compare transcranial direct current stimulation (tDCS) and tsDCS, which are associated with the stimulation of the primary motor cortex. Some authors found no correlation between the effects of stimulation and the intensity of the stimulation. The effects of the stimulation parameters on the physiological effects are partially non-linear [5]. For example, Jamil et al. systematically assessed the effects of four tDCS intensities of 0.5, 1, 1.5, and 2 mA applied for 15 min on cortical motor excitability and found no significant differences in the effects caused by these intensities on the anodic tDCS. The effects of lower intensities (0.5 and 1.0 mA) had an equal, if not greater, effect on motor excitability [6]. In addition to the intensity of tDCS, the duration of stimulation also affects the ability to induce a persistent increase in cortical excitability [7]. When the duration of 1 mA anodal tDCS doubled from 13 min to 26 min, the stimulation caused a decrease in excitability, which shows the dependence of the direction of the effects caused by the duration of tDCS [8]. Studies with a lower current (≤1 mA) and a shorter duration (≤13 min) reported an intensity- and duration-dependent effect of anodic tDCS, where the corresponding increase in parameters led to a linear dose–response relationship [7,9]. In particular, for anodal tDCS, increasing the current from 1 mA to 2 mA did not lead to a corresponding increase in MEP amplitude [6,10]. In addition, several studies have reported no change in MEP amplitude, especially with tDCS, in healthy populations [11], without additional or simultaneous motor training [12].

When tDCS and tsDCS are compared, there may be other differences in the effects of these two methods due to the different anatomical and functional constitutions between the cerebral cortex and the spinal cord. Nevertheless, a similar situation was observed when using tsDCS. There is a wide range of effects depending on small changes in the stimulation protocol. Some studies have shown that an increasing current does not necessarily lead to longer-lasting neuroplastic changes or treatment outcomes [5,8,13].

It was previously shown that tsDCS at the level of the cervical region (C7) with a current of 2.0 mA for 20 min increased the amplitude of MEP (flexor carpi radialis (FCR)) up to 2 h after stimulation, while the values of cathodal and anodal stimulations did not significantly differ from each other [14].

There are also contradictory data showing that tsDCS, both cathodal and anodal, at the cervical level (3 mA, 20 min, C6-T1, biceps brachii, flexor carpi radialis) [15] and with a parallel electrode configuration (2.5 mA, 15 min, C3-T3, abductor digiti minimi (ADM)) [16] with the declared parameters, does not change the MEP amplitude. This raises interest in finding more suitable tsDCS parameters with responses in the upper extremity muscles. For example, with direct current stimulation of the cervical spine, it was shown that the stimulation increased the MEP [17]. In another study, stimulation had no effect on motor responses, and, at the same time, the MEP amplitude did not change during tsDCS in either the simulation group or the sham group [14,15]. 

The current study focused on expanding information on the optimally appropriate stimulation current intensities to modulate CSS excitability and the ability of tsDCS to influence motor skills in healthy adults. In addition, the purpose of this study was to compare the new data obtained from the anodic cervical tsDCS with a current of 2.5 mA and the tsDCS data obtained earlier under the same conditions with a current of 1.5 mA.

## 2. Materials and Methods

### 2.1. Participants

Fifty-four healthy volunteers were enlisted through social media between January and June 2022, with a mean age of 21.19 ± 3.2 years. Criteria for exclusion encompassed a consistent sleep duration of less than 6 h per day, self-reported left-handedness, a history of brain injury or head trauma, diagnosed psychiatric or neurological conditions (including epilepsy and migraines), a family history of epilepsy, the use of prescribed medications, and the presence of metal objects within the body. The experiment was conducted in two separate stages, with 24 participants in the first stage and 30 participants in the second stage. In each stage, participants were evenly distributed into two groups. The first group underwent tsDCS. The second group received sham stimulation.

### 2.2. Experimental Design

The first stage of the study consisted of four blocks:

1. Conducting transcranial magnetic stimulation (TMS) to record 20 MEPs before stimulation (Tbefore);
2. Applying tsDCS (two distinct groups—anodal stimulation and sham) for 11 min;
3. Recording 20 MEPs immediately after the tsDCS session (T0) using TMS;
4. Conducting TMS to record 20 MEPs at a 15 min interval after the tsDCS session (T15).

**Figure 1:**
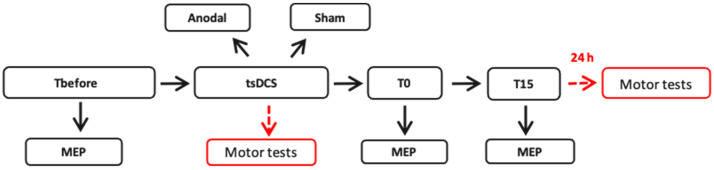
The experimental design, consisting of two stages, each comprising four blocks. Initially, a “hot spot” search was performed for the right FDI muscle, followed by the recording of 20 MEPs induced by TMS (Tbefore). Subsequently, participants underwent 11 min of either anodal tsDCS or sham. Following the stimulation, two blocks of MEP recordings were performed: the first immediately after tsDCS (T0) and the second 15 min post-stimulation (T15). In the second stage of the study, the subjects were divided into anodal and sham groups, undergoing similar tsDCS. However, during the 11 min tsDCS session, subjects performed two motor tests—the nine-hole peg test and the serial reaction time test—aimed at evaluating manual dexterity. The impact of tsDCS was evaluated by comparing the MEP ratios before and after stimulation at both immediate and 15 min post-tsDCS intervals in both stages.

The second stage of the study (Figure 1) replicated all these blocks from the first stage. However, during the stimulation phase, participants underwent motor tests, which were then repeated on the following day.

#### 2.2.1. Transcranial Magnetic Stimulation 

TMS was conducted utilizing the MagPro X100 MagVenture (MagVenture A/S, Farum, Denmark) transcranial magnetic stimulator, and the navigation of stimulation was facilitated by the Localite TMS Navigator system. The positioning, orientation, and contact of the TMS coil with the subject’s head were controlled using the Axilum Robotics TMS-Cobot robotic system, operated through an optical tracking system. This setup ensures a guaranteed error of less than 0.1 mm to target the same point in the brain consistently. TMS was applied to the “hot spot” of the right FDI muscle in M1, employing single biphasic impulses at 115 of motor resting.

#### 2.2.2. Transspinal Direct Current Stimulation 

**Figure 2:**
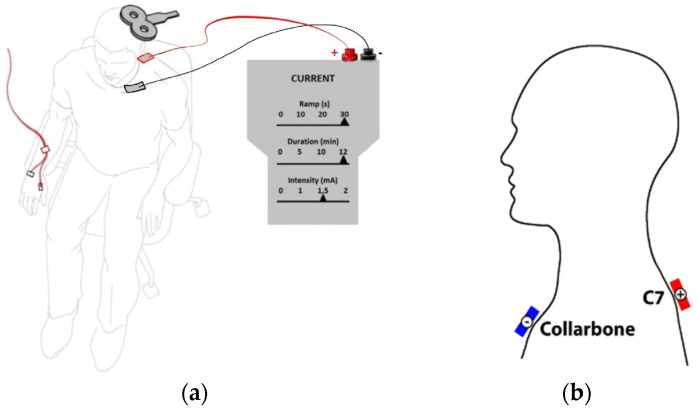
Position of electrodes. (**a**) General layout of TMS and tsDCS stimulating systems. Layout of electrodes: the anodal electrode was located above the C7-Th1 segment (red) and (**b**) the cathodal electrode was located on the clavicle (black).

tsDCS was administered using the BrainStim (EMS, Montecchio Emilia, Italy) system, with a current of 2.5 mA per electrode and a maximum voltage of 15 V. During the session, an anodal electrode measuring 5 × 5 cm was positioned at the level of the cervical spine (segmenta cervicalia C7-Th1), identified through palpation and anatomical landmarks. Simultaneously, a cathodal electrode measuring 5 × 10 cm was placed on the right clavicle (Figure 2). Before mounting the electrodes, gel (Uni Max; Geltek, Moscow, Russia) was applied for better electrical conductivity. The electrodes were securely affixed to the subjects’ skin using a patch before the 11 min stimulation session commenced. All procedures adhered to established guidelines [18].

#### 2.2.3. Electromyogram (EMG)

Electromyogram recording was performed using the BrainAmp EXG (Brain Products GmbH, Gilching, Germany) unit. EMG signals were filtered with a high-pass filter of 10 Hz and a notch filter of 50 Hz. Data were sampled at 1 kHz.

Surface electromyography (EMG) was recorded from the right FDI muscle. Electrode location: first dorsal interosseous muscle, musculus adductor pollicis, and ground electrode on the articulátio radiocarpea. The EMG recording lasted for 5 min, during which TMS-induced MEPs were recorded. The recording of electrical activity does not imply any interference in human activity. Only external manifestations of brain and muscle activity are recorded in the form of electrical currents, and this method does not pose any threat to human health.

#### 2.2.4. Motor Skill Tests

It is thought that tDCS enhances motor skill learning through the modulation of corticospinal excitability, which leads to the induction of long-term potentiation, a mechanism that underlies learning [9]. We hypothesized that if tsDCS also affects the excitability of the corticospinal system, as does tDCS, then perhaps transspinal stimulation would improve motor skill performance. A total of two sets of motor tests were performed. On day 1, during 11 min tsDCS, participants performed two types of motor tests (SRT and 9-HPT). The serial reaction time task (SRT) test measured the reaction time and accuracy, and the nine-hole peg test (9-HPT) measured the task completion time. Each participant had one attempt on the SRT and two attempts on the 9-HPT and the best time of the two attempts was selected. Both tests were carried out one after the other. On the second day, similar tests were performed without stimulation to evaluate tsDCS with motor skill performance, as motor memory consolidation occurs during resting and sleep. 

#### 2.2.5. Serial Reaction Time Task

The serial reaction time task (SRT) is a common measure of implicit learning. In this task, participants respond to a set of stimuli by pressing a button, unaware that probabilities are influencing the transitions between signals. Over time, participants learn and predict these transitions, leading to progressively faster response times.

#### 2.2.6. Nine-Hole Peg Test

**Figure 3:**
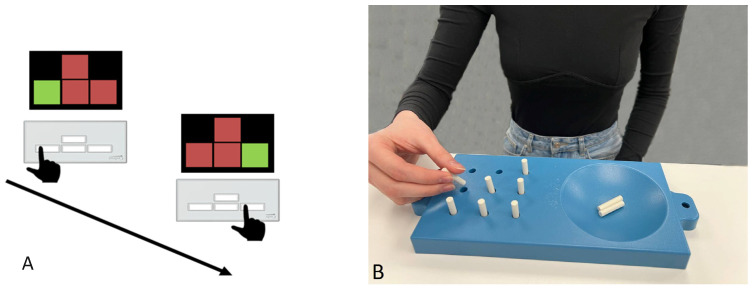
Used motor tests. (**A**) Layout of buttons in the serial reaction time task test. The subject must press the buttons located on the response pad in accordance with the image on the screen as quickly as possible. (**B**) Performing the nine-hole peg test. The subject must place the pegs in 9 holes as quickly as possible and fold them back as quickly as possible.

The nine-hole peg test assesses finger dexterity by recording the time taken to place pegs in designated holes, using a set of 9 pegs and a base plate with nine holes arranged in three rows (Figure 3). This test is employed to study fine motor skills in healthy individuals. 

### 2.3. Statistical Analysis

Statistical analysis was performed in R studio (R.1.0.) using the lmer package to build linear mixed-effect models. The modulation of MEPs was evaluated using a linear mixed-effect model. In particular, factor Group (df = 2, stimulation with current intensity 1.5 mA, 2.5 mA, and sham) and factor Time (df = 2, recordings performed either before tsDCS (Tbefore), immediately after tsDCS (T0), or 15 min after tsDCS (T15) were used as fixed effects, whereas the participants’ ID was used as a random intercept effect. The pre-stimulation MEP amplitudes of the stimulation group participants were used as a baseline condition for the model. 

Approximations of degrees of freedom for fixed effects were obtained by means of Satterthwaite approximation using the lmerTest package [19], following the recommendation by Luke et al. [20]. Considering that the main effect of the factors and their interaction was found to be significant, we acquired the estimated marginal means (EMMs) of pairwise comparisons for post hoc testing using the emmeans package for R [21]. The resulting *p*-values of the pairwise comparisons were corrected with respect to the false discovery rate (FDR) according to Benjamini and Hochberg’s adjustment [22].

Further, the time required for each participant to finish the motor task (performance timing) was fitted to mixed-designed repeated-measures ANOVA models with factor Group (df = 1; either S.0.0 or S.1.5) and factor Day (df = 1, either First or Second). 

In the current study, we performed a statistical comparison of our previous data with 1.5 mA tsDCS obtained using the same experimental design reported earlier [17].

## 3. Results

### 3.1. Effects of 1.5 mA

In our previous studies, we showed that the use of 11 min anodal tsDCS at the level of the cervical spine C7-Th1 with a current of 1.5 mA has an effect in terms of changing the excitability of the corticospinal system, which is expressed as a change in the amplitude of the MEP of the FDI muscle. First, the amplitude of the MEP induced by TMS decreases immediately after stimulation, but, 15 min after stimulation, the MEP amplitude increases, which was confirmed by statistical analysis. In addition, our previous data showed that anodal tsDCS with such a set of parameters does not affect the production of motor skills. The analysis was repeated in the current study. 

### 3.2. Effects of tsDCS on MEP Amplitudes

We examined the significance of the main effects (Group and Time) and interactions within the linear mixed-effect model, combining data collected during 1.5 mA stimulation (previously reported by Pomelova et al., 2022 [17]), 2.5 mA stimulation, and sham stimulation. In turn, it was shown that neither factor Group (F (2, 76) = 1.67, *p* = 0.19) nor factor Time (F (2, 4655) = 0.99, *p* = 0.37) explained the data significantly. However, the effect of their interaction (F (4, 4655) = 5.57, *p* < 0.001) was found to be significant. In particular, the linear mixed model fit of the MEP amplitudes recorded before (Tbefore), immediately after (T0), and with a 15 min delay after the stimulation or sham session (T15) was performed by restricted maximum likelihood (REML criterion at convergence = 26554.5). 

**Figure 4:**
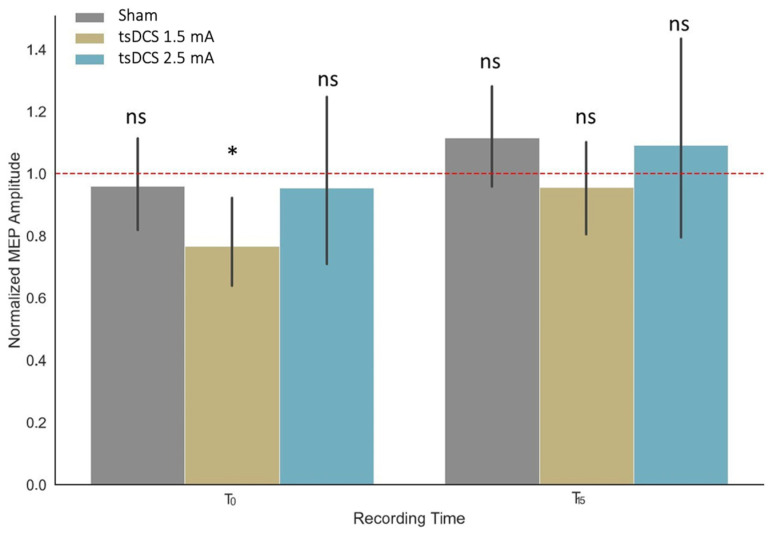
MEP amplitudes normalized by pre-stimulation (Tbefore) values for groups receiving tsDCS (blue) and sham (gray) stimulation. The MEPs were recorded immediately after the stimulation (T0) and with a 15 min delay (T15). Markers above the columns indicate the statistical significance of the estimated marginal mean difference between corresponding MEP amplitudes before and after the tsDCS/sham session (* *p* < 0.05; ns: *p* > 0.05). Error bars represent the 95% CI of the estimates.

We further conducted a set of pairwise comparisons between the estimated marginal means between sets of MEP aptitudes within one day, which showed no significant deviation from the baseline condition for all the pairwise comparisons, except for the comparison of baseline and T0 for the participants receiving 1.5 mA anodal stimulation (Figure 4).

We also compared the effects of stimulation during motor training and sham stimulation. No significant differences were observed (*p* = 0.457 F = 0.586).

### 3.3. Effect of tsDCS on the Development of New Motor Skills in Healthy Subjects

**Figure 5:**
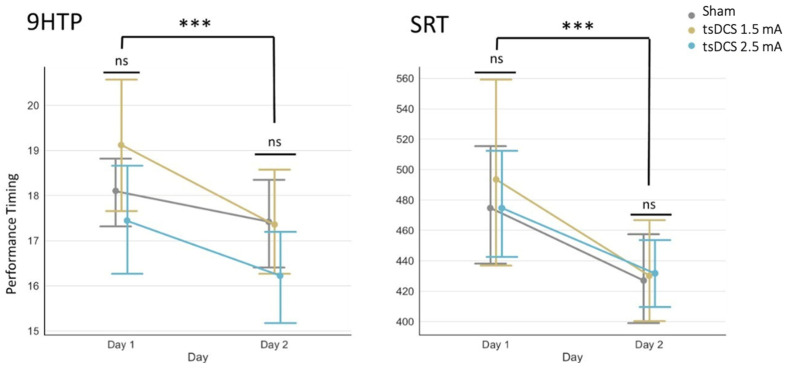
Performance timing of participants receiving 1.5 mA anodal tsDCS (yellow), 2.5 mA anodal tsDCS (blue), and sham (gray) stimulation assessed separately for the nine-hole peg test (9-HTP) and the serial reaction time task (SRT). No significance was observed for the Group factor, indicated by the special symbols between bars (ns: *p* > 0.05). Special symbols between groups of bars indicate the significance of the Day factor in each of the two ANOVA models (*** *p* < 0.001).

The effects of tsDCS stimulation on the development of new motor skills were assessed using the 9-HPT and SRT. The results of the ANOVA modeling showed that participants spent significantly less time finishing both motor tasks during the first-day experimental session, regardless of their group (Figure 5). 

In particular, for the 9-HTP task, the Group factor did not explain the variance in the data significantly (F(2, 39) = 0.083, *p* = 0.92), similar to the Group x Day interaction (F(2, 39) = 0.682, *p* = 0.51), whereas the effect of the Day factor was shown to be significant (F(1, 39) = 46.98, *p* < 10^−7^).

Similarly, for the SRT task, the Group factor did not explain the variance in the data significantly (F(2, 37) = 1.510, *p* = 0.23), nor did the Group x Day interaction (F(2, 37) = 1.711, *p* = 0.19), whereas the effect of the Day factor was shown to be significant (F(1, 37) = 24.00, *p* < 10^−4^).

## 4. Discussion

This study evaluated the effect of anodal tsDCS at the level of the cervical enlargement of the spinal cord at an intensity of 2.5 mA compared with 1.5 mA on CSS excitability and motor skills correction in healthy people. This protocol was also compared with our previously obtained data on the effect of anodal tsDCS at the level of the cervical enlargement of the spinal cord at an intensity of 1.5 mA. Data have been published [17], but no direct comparison with 2.5 mA stimulation has been made.

Our data show that tsDCS with these parameters affects neither the excitability of CSS nor the upper limb muscles. Moreover, the MEP amplitude of the FDI muscle induced by TMS in M1 changed neither after stimulation nor 15 min after stimulation.

Previously, we obtained data showing that the use of anodal tsDCS at the level of the cervical thickening of the spinal cord with an intensity of 1.5 mA changed the MEP amplitude after stimulation. The MEP amplitude decreased after stimulation and increased after 15 min, which indicates that tsDCS with an intensity of 1.5 mA effects a change in the excitability of the CSS [17]. Our studies show that changing even a single parameter, such as the stimulation intensity, can change the effect on CSS excitability, and that an increasing intensity does not always lead to a more pronounced effect. Although anodal tDCS typically increases the CSS excitability and MEP amplitude [23,24,25], (some studies suggest that the effect of tDCS may be absent or reversed [23,24,25]. The stimulation parameters [5], simultaneous peripheral stimulation [26], participant activity [27], and their individual characteristics [28,29] may influence the tDCS results.

Similar data were shown in the study of focal stimulation using a high-resolution tDCS anode. Stimulation at 1.5 mA was shown to immediately improve the movement time after stimulation in a motor test compared to a pre-motor test, and 2 mA reduced the learning effects, as did the sham [18]. However, some studies have shown that there is little to no difference in motor cortex excitability at tDCS intensities between 0.5 and 3 mA. However, a trend toward greater cortical excitability was observed at higher current strengths (1 vs. 3 mA) [30].

In another study, corticospinal excitability increased significantly after 0.7 mA anodal tDCS; however, the expected effect decreased and even vanished at intensities of 1 and 1.5 mA [31]. It is hypothesized that increasing the stimulation intensity with a constant stimulation duration activates counter-regulatory mechanisms to prevent the overexcitation of the brain.

Moreover, when comparing cervical tsDCS and tDCS studies, similar effects can be observed. The results of the Lim and Shin study of cervical tsDCS with an anteroposterior electrode configuration at an intensity of 2 mA for 20 min (recorded the MEP of the flexor carpi radialis muscle) showed that this stimulation, regardless of the stimulating electrode, affected the excitability of the CSS and caused an increase in the amplitude of the MEP, which lasted 2 h [14].

Dongés and D’Amico demonstrated that the application of 20 min cervical tsDCS at 3 mA using an anterior–posterior electrode configuration did not alter the muscle response (flexor carpi radialis and first dorsal interosseous) of the upper limbs for TMS [15].

Pereira et al. demonstrated that the application of tsDCS at the L2 level of the spine (2.5 mA), regardless of the direction of the current (anodal, cathodal, sham stimulation), did not affect the MEP of the abductor hallucis muscle [32]; the stimulation effects were consistent with the computer model. A possible explanation for our results may be the different electrophysiological properties of the spinal cord’s motor neurons, sensory neurons, and interneurons. One property to consider is polarization, which depends on the cellular orientation. Since interneurons have different orientations, the possible effects of tsDCS may rely mainly on the orientation of motor and sensory neurons in the spinal cord. In unidirectional synapses (such as the Ia-MN synapse), anode–cathode polarity can produce opposite effects due to the insufficiency of the electrical field generated in the anterior horns of the spinal cord.

The modeling the distribution of electron fields may be an effective method for a comparison of different montages and the determination of the optimal one for the electrode montage that maximizes electric field delivery during tsDCS [16].

The neuromodulator effects of tsDCS may result from local variations in the current density and electric field along neurons, resulting in specific polarization effects in the transmembrane potential, with axon endings being identified as the dominant cellular targets [16,33]. These variations are affected by various stimulation parameters, such as the placement and geometry of the electrodes or the intensity and polarity of the injected current, as with tDCS [34,35]. However, in comparison with the modeling of such fields during transcranial electrical stimulation, we encountered great difficulties—namely, the specificity of the bone pattern, the nonlaminar structure of the spinal cord, and many adjacent muscles and nerves. The results of the electrical simulations were also mixed. It may be necessary to accumulate data on different protocols to understand which method of tsDCS treatment of the spinal cord is the most effective. It is crucial to consider the duration of the effects in our data—the MEP response curve changes direction depending on the time after the end of the stimulation.

We also evaluated the effect of tsDCS on motor skills using the hand dexterity test (9-HTP) and the sequential reaction time test (SRT). Our data show that tsDCS at 2.5 mA does not affect the motor skills of subjects in these tests; the learning effects are similar during sham stimulation.

The use of tsDCS with an intensity of 1.5 mA did not affect motor skills or changes in MEP amplitude. It is possible that the performance of motor tasks during stimulation neutralizes the effect of the stimulation and that there is no effect on the excitability of the CSS.

There is no unequivocal opinion on whether it is worth conducting motor training before, during, or after stimulation. Studies have shown that it is more efficient to conduct motor training after stimulation, and the stimulation effect is maintained for 2 h, reducing fatigue in healthy subjects. This study examined motor skills in jumping [36]. The data also demonstrated that when performing step training with one-time stimulation, the acquisition and retention of motor skills in healthy subjects improved [37].

Many questions about the mechanisms underlying the neuromodulation of tsDCS spinal circuits remain unanswered, including the molecular basis and the neural targets involved in tsDCS (synaptic vs. intrinsic, interneurons, and motor neurons). Modeling and experimental studies have shown that spinal nerve roots and spinal neurons, particularly motor neurons [38], play a role in increasing motor activity using polarization through spinal neuromodulation [39,40].

tsDCS interacts with two functional neural circuits [41] that modulate the inhibitory pathway [42]. It can also activate the supraspinal loops that are transmitted through the brainstem or thalamo-cortical structures, with the subsequent inhibition of both ascending and descending pathways [43]. 

Inactive motor neurons may be activated after using tsDCS, which improves the connection between two functional neural circuits (cortical and spinal levels), and noticeable changes may occur in spinal motor neurons [44]. In addition, tsDCS is dependent on the polarity; therefore, researchers have found that cathodal stimulation activates neurons, while anodal stimulation depresses neurons [38].

Therefore, changing the current strength in the tsDCS protocol can lead to different results, interfering with CSS excitability. The protocol with the location of the anode electrode at the level of the seventh cervical vertebra and the cathode electrode on the collarbone is more effective and affects the excitability of the CSS at a current of 1.5 mA. However, there are no changes in the excitability of the CSS at a current of 2.5 mA (which is consistent with some of the available data) [14,15]. In addition, performing motor training during a stimulation session may neutralize the stimulation effect, and motor skills will not be corrected. Our study shows that anodal cervical tsDCS affects CSS excitability, but further research is needed to find the most effective protocols. 

### Limitations of the Study

Our study had several limitations. We recorded changes from only one muscle, which cannot indicate the functioning of the entire system. We also did not consider the effect of tsDCS on the peripheral nervous system, and we did not study the H-reflex. The F-wave, which is highly sensitive to changes in the excitatory state of the spinal cord, should also be explored. It is also still being determined precisely when it is best to conduct motor training: before tsDCS, after, or during.

## 5. Conclusions

(1) The use of 11 min anode tsDCS at the level of the cervical spine C7-Th1 with a current value of 2.5 mA does not cause a change in the amplitude of the MEP of the muscle of the upper limbs, in contrast to the current value of 1.5 mA, which affects the muscle of the upper limbs, first decreasing the amplitude of the MEP induced by TMS immediately after stimulation. Fifteen minutes after stimulation, the MEP amplitude increases.
(2) The application of tsDCS at the level of the upper spinal cord segments (C7-Th1) for 11 min at 2.5 mA and with a current strength of 1.5 mA does not affect the development of new motor skills in healthy people in the 9-HPT and SRT.

## Data Availability

Data is contained within the article.
